# GR, Sgk1, and NDRG1 in esophageal squamous cell carcinoma: their correlation with therapeutic outcome of neoadjuvant chemotherapy

**DOI:** 10.1186/s12885-020-6652-7

**Published:** 2020-02-27

**Authors:** Shunsuke Ueki, Fumiyoshi Fujishima, Takuro Kumagai, Hirotaka Ishida, Hiroshi Okamoto, Kai Takaya, Chiaki Sato, Yusuke Taniyma, Takashi Kamei, Hironobu Sasano

**Affiliations:** 1 Department of Gastrointestinal Surgery, Tohoku University Graduate School of Medicine, 1-1 Seiryo-machi, Aoba-ku, Sendai, 980-8574 Japan; 2 Department of Pathology, Tohoku University Graduate School of Medicine, 1-2 Seiryo-machi, Aoba-ku, Sendai, 980-8574 Japan

**Keywords:** Esophageal squamous cell carcinoma, Neoadjuvant chemotherapy, Glucocorticoid receptor, Sgk1, NDRG1, Chemo-resistance

## Abstract

**Background:**

Esophageal squamous cell carcinoma (ESCC) is a highly malignant neoplasm.

The glucocorticoid (GC)-glucocorticoid receptor (GR) pathway plays pivotal roles in cellular response to various stresses of tumor cells, including chemotherapy. However, the status of the GC-GR pathway in ESCC, including its correlation with chemotherapeutic responses, is largely unknown.

**Methods:**

GR, serum-and glucocorticoid-regulated kinase 1 (Sgk1), and N-myc down regulation gene 1 (NDRG1) were immunolocalized in 98 patients with ESCC who had undergone esophagectomy following neoadjuvant chemotherapy (NAC) with 2 courses of 5-fluorouracil + cisplatin. We also examined biopsy specimens before NAC in 42 cases and compared the results between those before and after NAC.

**Results:**

Overall survival (OS) of the patients treated with surgery following NAC was significantly shorter in the group with high GR than that with low GR status (*P* = 0.0473). Both OS and disease-free survival (DFS) were significantly shorter in both Sgk1- and NDRG1-high groups than in the low groups (OS: Sgk1, *P* = 0.0055; NDRG1, *P* = 0.0021; DFS: Sgk1, *P* = 0.0240; NDRG1, *P* = 0.0086). Biopsy specimens before NAC showed significantly shorter DFS in the high Sgk1 group (*P* = 0.0095), while both OS and DFS were shorter in the high NDRG1 group (OS, *P* = 0.0233; DFS, *P* = 0.0006) than in the respective low groups. In the high NDRG1 group of biopsy specimens before NAC, the tumor reduction rate by NAC was significantly attenuated (*P* = 0.021).

**Conclusions:**

High GR, Sgk1, and NDRG1 statuses in ESCC after NAC was significantly associated with an overall worse prognosis, with no significant changes in their expression levels before and after NAC. Therefore, increased activity of the GC-GR pathway with enhanced induction of Sgk1 and NDRG1 in carcinoma cells play pivotal roles in tumor progression and development of chemo-resistance in patients with ESCC undergoing NAC.

## Background

Esophageal cancer is the eighth most common human malignancy and the sixth most common cause of cancer deaths worldwide [1]. The standard treatment consists of surgical resection of locally advanced esophageal squamous cell carcinoma (ESCC) following neoadjuvant chemotherapy (NAC) [2]. However, the therapeutic effects of chemotherapy widely vary among cases, and satisfactory therapeutic outcomes cannot be obtained in many patients who received NAC [3, 4]. Therefore, predicting the therapeutic effects of NAC before starting therapy would enable the patients to avoid unnecessary chemotherapy and its clinical complications before surgery. Therefore, new therapeutic modes, as well as novel surrogate markers for predicting the therapeutic efficacy of NAC, are currently required for patients with locally advanced ESCC.

The glucocorticoid (GC)-glucocorticoid receptor (GR) signal pathway is well known to play pivotal roles in cellular response to various stresses [5]. This pathway was also reported to be involved in the stress response of tumor cells and simultaneously reduce the effects of chemotherapy by enhancing cellular response to stress in various carcinoma cells [6]. However, the correlation between the activity of the GC-GR pathway and the effects of chemotherapy has not been reported in ESCC.

GR is one of the members of the nuclear receptor superfamily. It binds to its ligand (GC), moves into the nucleus, and regulates the expression of GR-related genes including serum-and glucocorticoid-regulated kinase 1 (Sgk1), resulting in anti-inflammatory effects and improving cell survival [7]. In addition, GR was also reported to be more abundant in squamous cell carcinoma than in other histological types of cancer [8, 9]. Sgk1, a member of the glucocorticoid-responsive protein kinase family, is one of the major downstream markers of the GC-GR pathway and is regulated by steroids, p53, growth factors, and multiple other factors such as DNA damages, cell contraction, and oxidative stress [10–13]. Sgk1 is also known to regulate target genes, including N-myc down regulation gene 1 (NDRG1), which affects many physiological processes such as cell proliferation, differentiation, and apoptosis [14–16]. Sgk1 phosphorylates downstream NDRG1 and is involved in regulating tumor cell proliferation, differentiation, migration, and invasion [17]. However, the status of NDRG1 is also well known to be extremely varying between different carcinoma types. For instance, NDRG1 was reported to be downregulated in gastric [18] and colon [19] adenocarcinoma but upregulated in oral and pharyngeal squamous cell carcinoma [20], cervical adenocarcinoma [21], hepatocellular carcinoma [22], and non-small cell lung carcinoma [23]. In ESCC, NDRG1 abundance in carcinoma cells was reported to be significantly associated with less pronounced tumor invasion [24]. NDRG1 was also recently reported to be symmetrically upregulated in carcinoma cells and associated with local progression and poor prognosis in the patients with ESCC [25].

However, the correlations among GR, Sgk1, and NDRG1 have not been studied simultaneously among the same ESCC cases. Therefore, in this study, we evaluated the GR, Sgk1, and NDRG1 status in ESCC before and after NAC, and analyzed the clinical courses of the patients to assess the therapeutic efficacy of NAC and prognosis of the disease. We then attempted to clarify the potential involvement of the GC-GR pathway and identify markers for predicting the therapeutic efficacy of NAC before its administration in patients with locally advanced ESCC.

## Methods

### Patients

In this study, 98 ESCC patients were examined, all of whom underwent radical esophagectomy and regional lymph node dissection following NAC, according to the Japanese Clinical Oncology Group 9907 (JCOG9907) protocol at Tohoku University Hospital (Sendai, Japan) from April 2008 to December 2015 [3]. Among these 98 patients, biopsy specimens obtained prior to NAC were available in 42 cases. The specimens had been fixed in 10% neutral formalin for 36–48 h at room temperature and then embedded in paraffin wax. The sections were histologically examined according to the Eighth Edition of the Union for International Cancer Control tumor, node, and metastasis classification system [26]. The survival time of the patients was determined from the date of surgery until death, recurrence, or last censor. The current study protocol was approved by the Ethics Committee of the Tohoku University School of Medicine (Accession No. 2017–1-630), and informed consent was obtained from all patients prior to surgery.

### NAC and esophagectomy

Preoperative chemotherapy was performed according to the JCOG 9907 protocol [3] as follows: continuous infusion of 80 mg/m^2^ of cisplatin on days 1 and 22 and 5-fluorouracil (5-FU) 800 mg/m^2^/day, 24 h per day on days 1–5 and 22–26. In addition, 29.7 mg dexamethasone was administered per course to prevent the potential side effects of chemotherapy.

The therapeutic effects of preoperative chemotherapy were evaluated according to the Response Evaluation Criteria in Solid Tumors (RECIST) version 1.1 [27]. The patients were tentatively classified according to the new guidelines for determining the therapeutic effects of the solid tumors as complete response (CR), partial response (PR), progressive disease (PD), and stable disease (SD) [27]. According to the evaluation method reported in the JCOG 9907 protocol, the sum of the maximum diameter of the primary lesion and shortest diameter of lymph node lesions exceeding 1.5 cm were measured before and after treatment [3]. The maximum diameter of the primary lesion in CT following NAC corresponded to the slice measured by CT before treatment [3]. CR was defined as disappearance of the primary lesion, PR as reduction by 30% or more in maximum diameter of the primary lesion, PD as increase by 20% or more in maximum diameter of the primary lesion, and SD as other than CR, PD, and PR. A total of 13 cases were excluded from further evaluation because of difficulties in obtaining these parameters of clinical measurement. Histopathological tumor regression was tentatively classified into the following five categories according to the Japanese Classification of Esophageal Cancer, eleventh edition: grade 3, markedly effective (no viable residual tumor cells); grade 2, moderately effective (less than one-third residual tumor cells); grade 1, slightly effective (1b, one-third to two-thirds residual tumor cells; 1a, more than two-thirds residual tumor cells); grade 0, ineffective (no therapeutic effects detected) [28].

For esophagectomy, thoracoscopic esophageal subtotal excision, gastric tube reconstruction by hand-assisted laparoscopic technique or open laparotomy, and cervical esophagogastric anastomosis were performed with regional lymph node dissection.

### Immunohistochemistry

Serial tissue sections of 4-μm thickness, containing the deepest area of the tumor invasion, were deparaffinized in xylene, rehydrated in graded alcohol, and immersed in 3.0% hydrogen peroxide in methanol for 10 min at room temperature to inhibit endogenous peroxidase activity. For antigen retrieval, the tissue slides for GR, Sgk1, and NDRG1 immunohistochemistry were heated in an autoclave at 121 °C for 5 min in 0.01 M citrate buffer (pH 6.0). After washing three times for 5 min each in phosphate-buffered saline (PBS), the reacted slides were incubated in 1% normal goat serum for 30 min at room temperature to reduce nonspecific antibody binding and then incubated at 4 °C overnight with rabbit monoclonal antibody against GR (D6H2L, Cell Signaling Technology, Danvers, MA, USA, diluted 1/400), Sgk1 (Y238, Abcam, Cambridge, UK, diluted 1/200), or NDRG1 (EPR5593, Abcam, diluted 1/400). The reacted sections were then washed three times for 5 min each in PBS, incubated with biotinylated anti-rabbit immunoglobulin (Nichirei Biosciences, Inc., Tokyo, Japan), washed three times for 5 min each in PBS, and incubated with peroxidase-labeled streptavidin (Nichirei Biosciences, Inc.) for 30 min at room temperature. Immunoreactivity was visualized with 3,3^′^-diaminobenzidine, and the slides were counterstained with Mayer’s hematoxylin, dehydrated in graded alcohol, and cleared in xylene.

### Evaluation of immunoreactivity

GR immunoreactivity was evaluated in the nuclei of tumor cells and Sgk1 and NDRG1 in the cytoplasm of tumor cells. All immunostained slides were independently evaluated by two of the authors (SU and FF) without prior knowledge of any clinicopathological variables of the patients. GR immunoreactivity was semi-quantitatively assessed by H-score or calculating the percentage of nuclear-stained tumor cells multiplied by the relative immunointensity (0, negative; 1, weak; 2, moderate; 3, marked) resulting in a score in the range 0–300 [29]. Sgk1 and NDRG1 immunoreactivity was semi-quantitatively assessed by immunoreactive score, which was calculated as the percentage of cytoplasm-positive tumor cells (< 10%: 0, 10–25%: 1, 25–50%: 2, 50–75%: 3, 75–100%: 4) multiplied by the intensity of immunoreactivity (0: negative, 1: weak, 2: moderate, 3: marked) resulting in a score in the range 0–12 [23]. We determined the optimal H-score and immunoreactive score cut-off values for the survival outcome of the patients using the receiver operating characteristic curve method [30] and established thresholds of 154 for GR, 5 for Sgk1, and 7 for NDRG1.

A score in the range of 0–154 was tentatively considered as low GR, while that in the range of 155–300 as high GR. A score in the range 0–5 was also tentatively classified as low Sgk1, and 6–12 as high Sgk1. A score in the range 0–7 was tentatively determined as low NDRG1 and 8–12 as high NDRG1.

### Statistical analysis

JMP Pro version 13.2.0 software (SAS Institute, Inc., Cary, NC, USA) was used for all statistical analyses. Continuous data were analyzed using Student *t*-test or the Mann–Whitney U test. The relation and correlation between two variables were identified using the Pearson chi-square test, Fisher exact test, or Mann–Whitney U test and Wilcoxon test, as appropriate. Overall survival (OS) and disease-free survival (DFS) curves were constructed according to the Kaplan–Meier method and compared using the log-rank test. The Cox proportional hazard model was used for both univariate and multivariate analyses. When comparing paired pair values, the paired two-tailed *t*-test was used. A *P* value < 0.05 was considered statistically significant.

## Results

### Post-NAC status of GR, Sgk1, and NDRG1 and its correlation with clinicopathological variables in patients with ESCC

**Fig. 1:**
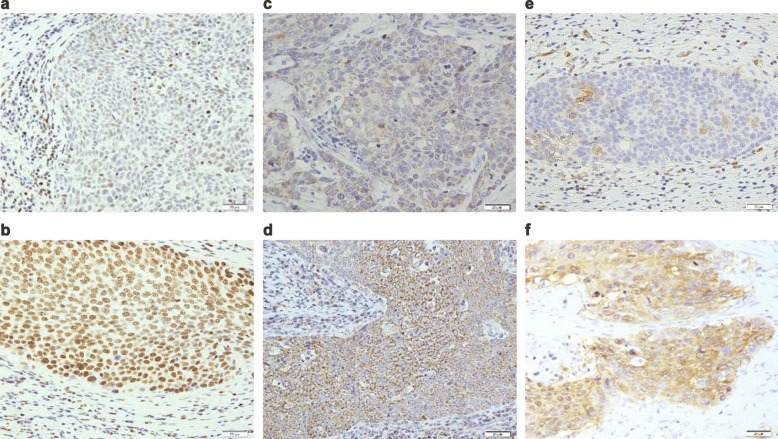
Representative illustrations of GR, Sgk1, and NDRG1 immunohistochemistry. **a** Low GR status and **b** High GR status; representative case showing diffuse and marked immunoreactivity in the nuclei of carcinoma cells. **c** Low Sgk1 status. **d** High Sgk1 status; representative case demonstrating Sgk1 immunoreactivity in the cytoplasm of carcinoma cells. **e** Low NDRG1. **f** High NDRG1; representative case demonstrating NDRG1 immunoreactivity in the cytoplasm and membrane of carcinoma cells

Representative micrographs showing GR, Sgk1, and NDRG1 expression are illustrated in Fig. 1. In surgical specimens following NAC, a high status of GR, Sgk1, and NDRG1 was detected in 53.1% (52/98), 54.1% (53/98), and 38.8% (38/98) of the patients, respectively (Table 1). The status of GR was significantly correlated with the presence of vessel invasion (*P* = 0.016), that of Sgk1 with pT (*P* = 0.006) and pN (*P* < 0.001), pStage (*P* < 0.001), lymphovascular invasion (*P* = 0.003), RECIST grade (*P* = 0.007), and histopathological tumor regression grade (*P* = 0.037). The NDRG1 status was significantly correlated with pT (*P* < 0.001), pStage (*P* = 0.006), and lymphovascular invasion (*P* = 0.033).

**Table 1:** post-NAC status of GR, Sgk1, NDRG1 and its correlation with clinicopathological variables

Variable		n	post-NAC GR expression		P	post-NAC Sgk1 expression		P	post-NAC NDRG1 expression		P
			High	Low		High	Low		High	Low	
Age	≧65y.o	62	32	30		35	27		22	40	
	< 65y.o	36	20	16	*p* = 0.706	18	18	*p* = 0.537	16	20	*p* = 0.380
Gender	Male	83	43	40		48	35		32	41	
	Female	15	9	6	*p* = 0.559	5	10	*p* = 0.080	6	9	*p* = 0.915
pT^a^	pT1a~pT1b	28	12	16		9	19		1	27	
	pT2~pT4b	70	40	30	*p* = 0.201	44	26	*p* = 0.006*	37	33	*p* < 0.001*
pN^a^	pN0	29	14	15		7	22		9	20	
	pN1~N3	69	38	31	*p* = 0.539	46	23	*p* < 0.001*	29	40	*p* = 0.308
cM^a^	cM0	94	50	44		49	45		36	58	
	cM1	4	2	2	*p* = 0.900	4	0	*p* = 0.060	2	2	*p* = 0.638
pStage^a^	pStageI,II	40	20	20		11	29		9	31	
	pStageIII,IV	58	32	26	*p* = 0.614	42	16	*p* < 0.001*	29	29	*p* = 0.006*
Tumor differentiation^a^	well,moderate	84	47	37		44	40		33	51	
	poor	10	4	6	*p* = 0.339	8	2	*p* = 0.097	5	5	*p* = 0.517
	Unclassifiable	4	1	3		1	3		0	4	
Lymphatic invasion	Absence	29	14	15		9	20		9	20	
	Presence	69	38	31	*p* = 0.538	44	25	*p* = 0.003*	29	40	*p* = 0.308
Vessel invasion	Absence	18	5	13		6	12		3	15	
	Presence	80	47	33	*p* = 0.016*	47	33	*p* = 0.051	35	45	*p* = 0.033*
RECIST grade^b^	CR/PR	28	12	16		10	18		8	20	
	SD/PD	57	34	23	*p* = 0.144	38	19	*p* = 0.007*	28	29	*p* = 0.072
	Indeterminate	13	6	7		5	8		2	11	
Histopathological tumor regression grade^c^	Grade0~1a	63	37	26		39	24		27	36	
	Grade1b~ 2	35	15	20	*p* = 0.131	14	21	*p* = 0.037*	11	24	*p* = 0.267
Total		98	52	46		53	45		38	60	

* Statistical significance

^a^ Tumor-node-metastasis (TNM) classification based on the 8th edition of the TNM classification of malignant tumors

^b^ New response evaluation criteria in solid tumors: revised RECIST guideline (version 1.1)

^c^ Histopathological features based on the Japanese Classification of Esophageal Cancer, 11th edition (Japan Esophageal Society 2015)

### Post-NAC status of GR, Sgk1, and NDRG1 in carcinoma cells and their correlation with patient survival

**Fig. 2:**
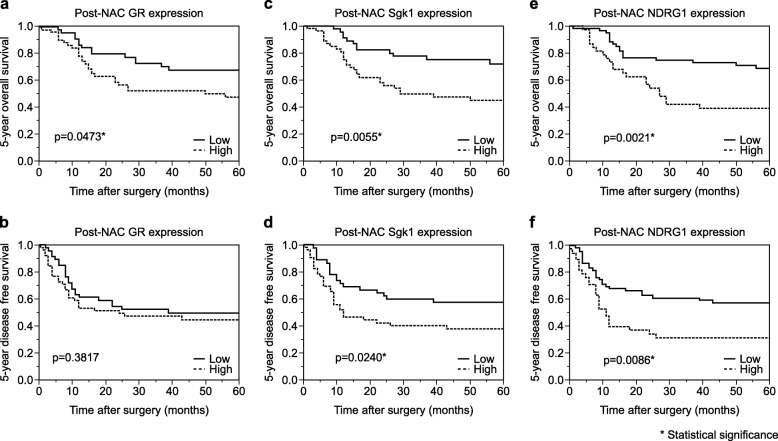
Kaplan–Meier curves post-NAC GR, post-NAC Sgk1, and post-NAC NDRG1. **a** The 5-year overall survival of the patients with esophageal squamous cell carcinoma (ESCC) exhibiting high post-NAC GR was significantly worse than those with low post-NAC GR. **b** No significant differences in the 5-year disease-free survival (DFS) detected between those exhibiting high and low post-NAC GR in carcinoma tissues. **c** The 5-year overall survival of those exhibiting high post-NAC Sgk1 was significantly worse than those of low post-NAC status. **d** The 5-year DFS of those exhibiting high post-NAC Sgk1 was significantly worse than those with low post-NAC Sgk1. **e** The 5-year overall survival of those exhibiting high post-NAC NDRG1 was significantly worse than those with low post-NAC NDRG1. **f** The 5-year DFS of those exhibiting high post-NAC NDRG1 was significantly worse than those with low post-NAC NDRG1

Five-year OS rate of the patients harboring high GR status was significantly shorter than those harboring low GR group (*P* = 0.0473) (Fig. 2a). In addition, significantly shorter 5-year OS and DFS were detected in the patients with high Sgk1 than in those with low Sgk1 (OS: *P* = 0.0055, DFS: *P* = 0.0240) (Fig. 2c and d). The 5-year OS and DFS were both significantly shorter in those with high NDRG1 than in those with low NDRG1 (OS: *P* = 0.0021, DFS: *P* = 0.0086) (Fig. 2e and f). Univariate analysis revealed that patient survival was significantly associated with pT (*P* = 0.0011) and pN (*P* = 0.0002), pStage (*P* = 0.0001), lymphatic invasion (*P* = 0.0260), vascular invasion (*P* = 0.0178), RECIST grade (*P* = 0.0015), histopathological tumor regression grade (*P* = 0.0432), high GR (*P* = 0.0479), high Sgk1 (*P* = 0.0054), and high NDRG1 (*P* = 0.0033) (Table 2). However, multivariate analysis demonstrated that pN was the only independent prognostic factor among all the variables examined (*P* = 0.0168) (Table 3).

**Table 2:** Univariable analysis of patients’ 5-year overall survival

Variable	P	Hazard ratio(95% CI)
Age (< 65/> 65)	0.9971	1.0011 (0.5156–1.8782)
Gender (female/male)	0.6125	1.2410 (0.5036–2.6421)
pT (pT1/pT2,3,4)	0.0011*	3.7983 (1.6271–11.083)
pN(pN0/pN1,2,3)	0.0002*	4.9747 (1.9864–16.656)
M (M0/M1)	0.5615	1.5686 (0.2551–5.1250)
pStage (pStageI,II/pStageIII,IV)	0.0001*	3.7464 (1.8595–8.3655)
Tumor differentiation^a^ (G1, G2/G3)	0.5702	1.3259 (0.4539–3.1015)
Lymphatic invasion (−/+)	0.0260*	2.3425 (1.0999–5.7744)
Vessel invasion (−/+)	0.0178*	3.3192 (1.1995–13.757)
Recist grade^b^ (PR, CR/SD, PD)	0.0015*	3.4976 (1.5642–9.3125)
Histopathological tumor regression grade^c^ (Grade0,1a/Grade1b,2)	0.0432*	0.4956 (0.2300–0.9795)
post-NAC GR expression (Low/High)	0.0479*	1.8991 (1.0060–3.7409)
post-NAC Sgk1 expression (Low/High)	0.0054*	2.5045 (1.3030–5.1191)
post-NAC NDRG1expression (Low/High)	0.0033*	2.5632 (1.3716–4.8540)

Abbreviation: *CI* confidence interval

* Statistical significance

**Table 3:** Multivariable analysis of patients’ 5-year overall survival

Variable	P	Relative risk (95% CI)
pT(pT1/pT2,3,4)	0.8106	1.1614 (0.3658–4.5669)
pN(pN0/pN1,2,3)	0.0168*	3.5661 (1.2375–13.083)
Lymphatic invasion (−/+)	0.9334	1.0437 (0.4128–3.2219)
Vessel invasion (−/+)	0.3204	2.0983 (0.5289–14.245)
Recist grade (PR,CR/SD, PD)	0.2774	1.7160 (0.6658–5.2041)
Histopathological tumor regression grade (Grade0,1a/Grade1b,2)	0.2314	0.5931 (0.2257–1.3705)
post-NAC GR expression (Low/High)	0.1524	1.6737 (0.8299–3.5502)
post-NAC Sgk1 expression (Low/High)	0.7961	0.9011 (0.4221–2.0594)
post-NAC NDRG1 expression (Low/High)	0.1184	1.7590 (0.8664–3.6655)

Abbreviation: *CI* confidence interval

* Statistical significance

### Correlation among post-NAC GR, Sgk, and NDRG1 in ESCC

A significant positive correlation was detected between post-NAC GR and Sgk1 or NDRG1 status in the tumor tissues (GR versus. Sgk1: *P* = 0.0084, GR versus. NDRG1: *P* = 0.0446). A significant positive correlation was also detected between post-NAC Sgk1 and NDRG1 status of the tumor tissues (*P* = 0.0009) (Table 4).

**Table 4:** Correlation of post-NAC GR, Sgk, NDRG status in carcinoma tissues

Variable	P	Correlation coefficient(95% CI)
post-NAC GR expression score (9–279) vs post-NAC Sgk1 score (0–12)	0.0084*	0.2647 (0.0699–0.4400)
post-NAC GR expression score (9–279) vs post-NAC NDRG1 score (0–12)	0.0446*	0.2034 (0.0052–0.3862)
post-NAC Sgk1 expression score (0–12) vs post-NAC NDRG1 score (0–12)	0.0009*	0.3316 (0.1426–0.4973)

Abbreviation: *CI* confidence interval

* Statistical significance

### Correlation of pre-NAC GR, Sgk1, and NDRG1 status with clinicopathological variables in ESCC patients undergoing NAC

In the biopsy specimens of ESCC patients prior to NAC, high GR, Sgk1, and NDRG1 were detected in 54.7% (23/42), 45.2% (19/42), and 42.9% (18/42) of the patients examined, respectively (Table 5). Among these, the pre-NAC GR status in carcinoma cells was significantly correlated with pStage (*P* = 0.037), and pre-NAC NDRG1 status was significantly correlated with RECIST grade (*P* = 0.021) in the patients following NAC.

**Table 5:** Pre-NAC status of GR, Sgk1, NDRG1 and its correlation with clinicopathological variables of the cases

Variable		n	pre-NAC GR expression		P	pre-NAC Sgk1 expression		P	pre-NAC NDRG1 expression		P
			High	Low		High	Low		High	Low	
Age	≧65y.o	26	15	11		11	15		12	14	
	< 65y.o	16	8	8	*p* = 0.627	8	8	*p* = 0.627	6	10	*p* = 0.581
Gender	Male	35	18	17		15	20		13	22	
	Female	7	5	2	*p* = 0.332	4	3	*p* = 0.489	5	2	*p* = 0.094
pT^a^	pT1a~pT1b	11	5	6		3	8		3	8	
	pT2~pT4b	31	18	13	*p* = 0.470	16	15	*p* = 0.156	15	16	*p* = 0.216
pN^a^	pN0	15	6	9		5	10		9	15	
	pN1~N3	27	17	10	*p* = 0.152	14	13	*p* = 0.246	12	15	*p* = 0.780
cM^a^	cM0	40	22	18		17	23		17	23	
	cM1	2	1	1	*p* = 0.890	2	0	*p* = 0.070	1	1	*p* = 0.835
pStage^a^	pStageI,II	17	6	11		4	13		5	12	
	pStageIII,IV	25	17	8	*p* = 0.037*	15	10	*p* = 0.020	13	12	*p* = 0.147
Tumor differentiation^a^	well,moderate	37	20	17		16	21		17	20	
	poor	4	3	1	*p* = 0.427	3	1	*p* = 0.220	1	3	*p* = 0.426
	Unclassifiable	1	0	1		0	1		0	1	
Lymphatic invasion	Absence	13	6	7		4	9		5	8	
	Presence	29	17	12	*p* = 0.453	15	14	*p* = 0.207	13	16	*p* = 0.700
Vessel invasion	Absence	8	6	3		4	5		4	5	
	Presence	34	17	16	*p* = 0.418	15	18	*p* = 0.957	14	19	*p* = 0.914
RECIST grade^b^	CR/PR	14	5	9		4	10		3	11	
	SD/PD	25	16	9	*p* = 0.089	14	11	*p* = 0.097	15	10	*p* = 0.021*
	Indeterminate	3	2	1		1	2		0	3	
Histopathological tumor regression grade^c^	Grade0~1a	27	17	10		14	13		14	13	
	Grade1b~ 2	15	6	9	*p* = 0.152	5	10	*p* = 0.246	4	11	*p* = 0.109
Total		42	23	19		19	23		18	24	

* Statistical significance

^a^ Tumor-node-metastasis (TNM) classification based on the 8th edition of the TNM classification of malignant tumors

^b^ New response evaluation criteria in solid tumours: revised RECIST guideline (version 1.1)

^c^ Histopathological features based on the Japanese Classification of Esophageal Cancer, 11th edition (Japan Esophageal Society 2015)

### Correlation of pre-NAC GR, Sgk1, and NDRG1 status in carcinoma tissues with the survival of ESCC patients undergoing NAC

**Fig. 3:**
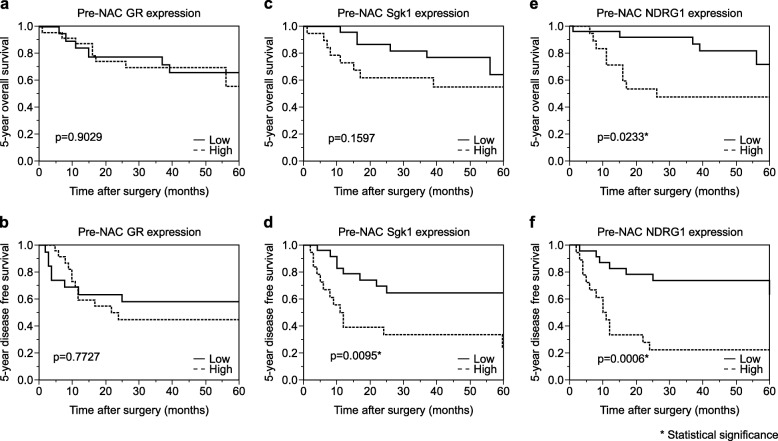
Kaplan–Meier curves pre-NAC GR, pre-NAC Sgk1, and pre-NAC NDRG1. **a** and **b** No significant difference in the 5-year overall survival(OS) and 5-year disease-free survival (DFS) were detected between those exhibiting high and low pre-NAC GR. **c** No significant differences in the OS were detected between those exhibiting high pre-NAC Sgk1 and low pre-NAC GR. **d** The 5-year DFS of those exhibiting high pre-NAC Sgk1 was significantly worse than those with low pre-NAC in carcinoma tissues. **e** The 5-year OS of those exhibiting high pre-NAC NDRG1 was significantly worse than those with low pre-NAC NDRG1. **f** The 5-year DFS of those exhibiting high pre-NAC NDRG1 expression was significantly worse than those with low pre-NAC NDRG1 in carcinoma tissues

There were no significant correlations between the pre-NAC GR status in carcinoma cells and the 5-year OS or DFS of ESCC patients (3A and B). However, a significantly shorter DFS was detected in those with high pre-NAC Sgk1 status compared to those with low status (*P* = 0.0095) (Fig. 3d). Significantly shorter OS and DFS were also detected in those with high pre-NAC NDRG1 status compared to those with low status (OS: *P* = 0.0233, DFS: *P* = 0.0006) (Fig. 3e and f).

### Changes in GR, Sgk1, and NDRG1 before and after NAC

We examined the changes in GR, Sgk1, and NDRG1 before and after NAC in 42 patients. The results are summarized in Table 6. The concordance rates before and after NAC were 69.0% (GR), 85.8% (Sgk1), and 73.8% (NDRG1), respectively. As summarized in Table 7, we also performed a paired two-tailed bilateral *t*-test for the scores before and after NAC. The results are as follows: GR (*t* = 1.597, df = 41, *P* = 0.1178), Sgk1 (*t* = 1.723, df = 41, *P* = 0.0924), and NDRG1 (*t* = 1.274, df = 41, *P* = 0.2097). There were no significant changes in these scores above before and after NAC.

**Table 6:** Summary of the expression status changes between before and after NAC

expression status change between pre and after NAC	GR(%)	Sgk1(%)	NDRG1(%)
same expression status^a^	29 (69.0%)	36 (85.8%)	31 (73.8%)
Increased expression status^b^	3 (7.1%)	3 (7.1%)	5 (11.9%)
Decreased expression status^c^	10 (23.9%)	3 (7.1%)	6 (14.3%)
Total cases	42	42	42

^a^ The Same expression status group consisted of cases that belonged to the high expression group before and after NAC, or cases that belonged to the low expression group before and after NAC

^b^ The increased expression status group consisted of cases that belonged to the low expression group before NAC and high expression group after NAC

^c^ The decreased expression status group consisted of cases that belonged to the high expression group before NAC and low expression group after NAC

**Table 7:** Summary of two-tailed t-test of the scores before and after NAC

Variable (score average)	t	df	P	Correlation coefficient (95% CI)
pre-NAC GR score (152.3) vs post-NAC GR score (141.8)	1597	41	0.1178	0.1922(−0.1186–0.4688)
pre-NAC SgK1 score (4.976) vs post-NAC Sgk1 score (4.333)	1.723	41	0.0924	0.7120 (0.5208–0.8352)
pre-NAC NDRG1 score (7.024) vs post-NAC NDRG1 score (6.357)	1.274	41	0.2097	0.3780 (0.0837–0.6117)

**Fig. 4:**
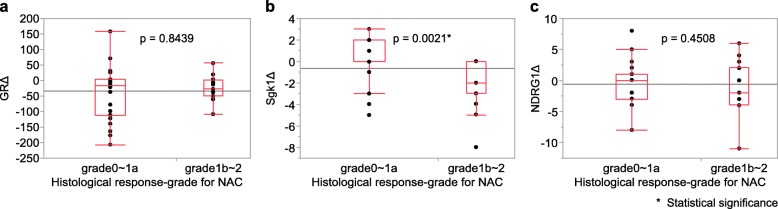
The correlation between the changes in scores (Δ) of GR, Sgk1, NDRG1 and histopathological tumor regression grade were examined using the Wilcoxon test. **a** GR (*P* = 0.8439), **b** Sgk1 (*P* = 0.0021), C) NDRG1 (*P* = 0.4508)

Figure 4 demonstrates the correlation between the changes in GR, Sgk1, and NDRG1 scores before and after NAC and the histopathological tumor regression grade. There were no significant associations between the changes in GR or NDRG1 scores before and after NAC and the histopathological tumor regression grade. However, the Sgk1 score significantly increased following NAC in patients with a low histopathological tumor regression grade (*P* = 0.0021).

**Fig. 5:**
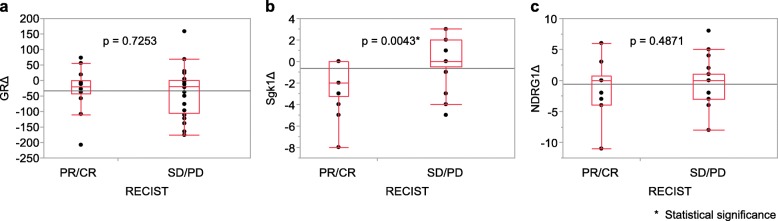
The correlation between the changes in scores (Δ) of GR, Sgk1, NDRG1 and RECIST grade were examined using the Wilcoxon test. **a** GR (*P* = 0.7253), **b** Sgk1 (*P* = 0.0043), **c** NDRG1 (*P* = 0.4871)

Figure 5 demonstrates the correlation between the changes of GR, Sgk1 and NDRG1 scores before and after NAC and RECIST grade of the patients examined in this study. There were no significant associations between the changes in GR or NDRG1 scores before and after NAC and RECIST grade but the Sgk1 score significantly increased after NAC in the SD/PD groups (*P* = 0.0043).

## Discussion

This is the first study to evaluate the status of GR, SgK1, and NDRG1 before and after NAC in ESCC patients. Notably, the high GR, Sgk1, and NDRG1 status in resected specimens were significantly associated with shorter OS in those undergoing NAC. A previous study reported no significant correlation between the GR status and the clinical outcome in ESCC patients [7]; however, in the current study, we comprehensively evaluated GR immunoreactivity using the H-score. In addition, we also evaluated the NAC cases in which the GC-GR pathway could be more activated. This may account for the discrepancy between these two studies, but further investigations are required for confirmation.

Sgk1 has not been studied in ESCC. We found that the Sgk1 status in carcinoma cells was not only significantly associated with shorter OS and DFS but also with more advanced pT, pN, and lymphatic vessel invasion in ESCC patients. Sgk1 is well known to activate beta-catenin/T cell factor signaling in human non-small cell lung cancer, and to be associated with tumor cell invasion and migration [31]. Therefore, Sgk1 could also enhance tumor cell invasion and migration of ESCC, as reported for other human malignancies such as esophagogastric junctional adenocarcinoma [32], colorectal cancer [33], and non-small cell lung carcinoma [31].

NDRG1 has been examined in ESCC [24, 25]. In our present study, a high NDRG1 status was significantly associated with shorter OS and DFS, higher pT, and local progression factors such as venous invasion in the patients, which is consistent with the results of previously reported studies [24, 25]. In addition, the Wnt pathway was also reported to be activated via NDRG1 and involved in epithelial-mesenchymal transition of ESCC tumor cells [25]. Results from our present study indicate that NDRG1 was activated via Sgk1 in ESCC undergoing NAC and thus could be involved in the local progression of the tumor.

We then examined the correlation between GR, Sgk1, and NDRG1 status before and after NAC and the clinicopathological factors of the ESCC patients. Differences were detected between post- and pre-NAC and this discrepancy is considered as a limitation of our present study as intratumoral heterogeneity was high and the number of biopsy specimens available for examination was rather small in this study. In addition, a high GR status in resected specimens, but not biopsy specimens, turned out as a poor prognostic factor. However, there were no significant changes in the GR score before and after chemotherapy. This may also be due to intratumoral heterogeneity but in breast cancer patients, steroid hormone receptor profiles were also reported to be different between pre- and post-chemotherapy [34], and similar changes in GR may occur in ESCC patients but further investigations are required for clarification.

Furthermore, GR, Sgk1, and NDRG1 were all significantly correlated with prognosis when evaluated by univariate analysis. However, the results of multivariate analysis demonstrated that these factors were not independent predictors of patient outcomes. This discrepancy may be because GR, Sgk1, and NDRG1 were significantly correlated with each other in the patients examined in this study. In addition, both Sgk1 and NDRG1 were significantly correlated with established clinicopathological factors such as pT and pN in patients.

In our present study, a significant positive correlation was detected among GR, Sgk1, and NDRG1 status in ESCC cases examined. Sgk1 is activated by growth factors, DNA damages, cell contraction, and oxidative stress, in addition to the GC-GR pathway [10–12]. NDRG1 is also known to be activated by stress signals, oxidative balance [35], DNA damages, increased p53 expression [36], and hypoxia [37], in addition to the GC-GR pathway. Therefore, Sgk1 and NDRG1 in ESCC carcinoma cells could be affected by various factors other than the GC-GR pathway, and further studies are required to clarify the GR-Sgk1-NDRG1 axis in ESCC.

In this study, we compared the GR, Sgk1, and NDRG1 status between ESCC before and after NAC to further clarify the significance of the GC-GR pathway in the therapeutic effects of NAC. A high NDRG1 status in carcinoma cells in pre-NAC biopsy specimens was significantly associated with lower NAC effects, and a high GR and Sgk1 status in carcinoma cells tended to be associated with lower NAC effect. In addition, a lower NAC effect was significantly associated with poor prognosis of ESCC patients examined. Therefore, these results suggest that NAC sensitivity was decreased in tumors with high expression of GR, Sgk1, and NDRG1, resulting in higher residual tumor cells and subsequent adverse clinical outcomes of patients. This also indicates that the efficacy of NAC could be predicted by analyzing the GR pathway.

We also examined whether a high NDRG1 status in biopsy specimens was significantly associated with decreased therapeutic effects of NAC in patients and observed no significant association. This may be due to the relatively small number of biopsy specimens. However, carcinoma tissues were not available for examination among resected specimens of ESCC patients who completely responded to NAC. In addition, the detailed molecular mechanisms underlying the GR-Sgk1-NDRG1 pathway-mediated resistance to NAC in ESCC patients has remained virtually unknown. Cell cycle arrest via the GC-GR pathway is well known to contribute to chemo-resistance in cancer patients [38]. Factors including Sgk1 have also been considered as the cause of decreased sensitivity of cytotoxic drug therapy by activating the GC-GR pathway in hepatocellular carcinoma and colon adenocarcinoma [39]. Besides, GR-mediated Sgk1 activation suppressed tumor cell apoptosis in breast carcinoma cells [40] and NDRG1 was reported to be induced by Sgk1 to inhibit apoptosis in ESCC tumor cells [41]. These results above, as well as those from our present study, did indicate that the GR-Sgk1-NDRG1 pathway in ESCC could protect tumor cells from chemotherapy-induced apoptosis and mediate chemotherapy resistance.

We examined the changes in various factors before and after NAC to explore the effects of NAC and synthetic steroids administered during NAC on GR, SgK1, and NDRG1 expression. In particular, the administration of synthetic steroids during NAC was reported to induce chemotherapy resistance in patients with urological [42] and breast cancers [43]. Therefore, steroid administration is considered to reduce treatment sensitivity. However, in this study, there were no significant differences of GR, Sgk1, and NDRG1 scores before and after NAC, although the Sgk1 score after NAC was significantly higher in patients with low NAC treatment effects than those without. Therefore, the therapeutic effects of chemotherapy may be reduced in the Sgk1 high group by stress stimulation or synthetic steroids administered during NAC administration. However, there were no significant associations between the changes in GR and NDRG1 scores before and after NAC and the therapeutic effects of the patients. Therefore, further studies are required to clarify the effects of NAC and synthetic steroids administered during NAC on the GR, Sgk1, and NDRG1 status.

## Conclusions

Our results suggest that the status of GR, Sgk1, and NDRG1 in ESCC patients undergoing NAC was significantly related to the treatment outcomes and that the GR-Sgk1-NDRG1 pathway in carcinoma cells of ESCC might be involved in the clinical effects of chemotherapy in these patients. Further, data from the study suggest the potential utility of GR, Sgk1 and NDRG1 as prognostic markers for ESCC patients undergoing NAC, though further validating study is needed.

## Data Availability

The datasets used and/or analyzed during the current study are available from the corresponding author on reasonable request.

## Abbreviations

5-FU: 5-Fluorouracil
CDDP: Cisplatin
DFS: Disease-free survival
ESCC: Esophageal squamous cell carcinoma
GC: Glucocorticoid
GR: Glucocorticoid receptor
NAC: Neoadjuvant chemotherapy
NDRG1: N-myc down regulation gene 1
OS: Overall survival
PBS: Phosphate-buffered saline
RECIST: Response Evaluation Criteria in Solid Tumors
Sgk1: Serum-and glucocorticoid-regulated kinase 1

## References

[1] Ferlay J, Soerjomataram I, Dikshit R, Eser S, Mathers C, Rebelo M, Parkin DM, Forman D, Bray F (2015). Cancer incidence and mortality worldwide: sources, methods and major patterns in GLOBOCAN 2012. Int J Cancer.

[2] Kitagawa Y, Uno T, Oyama T, Kato K, Kato H, Kawakubo H, Kawamura O, Kusano M, Kuwano H, Takeuchi H (2019). Esophageal cancer practice guidelines 2017 edited by the Japan esophageal society: part 1. Esophagus..

[3] Ando N, Kato H, Igaki H, Shinoda M, Ozawa S, Shimizu H, Nakamura T, Yabusaki H, Aoyama N, Kurita A (2012). A randomized trial comparing postoperative adjuvant chemotherapy with cisplatin and 5-fluorouracil versus preoperative chemotherapy for localized advanced squamous cell carcinoma of the thoracic esophagus (JCOG9907). Ann Surg Oncol.

[4] Medical Research Council Oesophageal Cancer Working Group (2002). Surgical resection with or without preoperative chemotherapy in oesophageal cancer: a randomised controlled trial. Lancet.

[5] Kadmiel M, Cidlowski JA (2013). Glucocorticoid receptor signaling in health and disease. Trends Pharmacol Sci.

[6] Oakley RH, Cidlowski JA (2013). The biology of the glucocorticoid receptor: new signaling mechanisms in health and disease. J Allergy Clin Immunol.

[7] Lin KT, Wang LH (2016). New dimension of glucocorticoids in cancer treatment. Steroids..

[8] Block TS, Murphy TI, Munster PN, Nguyen DP, Lynch FJ (2017). Glucocorticoid receptor expression in 20 solid tumor types using immunohistochemistry assay. Cancer Manag Res.

[9] Lien HC, Lu YS, Shun CT, Yao YT, Chang WC, Cheng AL (2008). Differential expression of glucocorticoid receptor in carcinomas of the human digestive system. Histopathology..

[10] Chen SY, Bhargava A, Mastroberardino L, Meijer OC, Wang J, Buse P, Firestone GL, Verrey F, Pearce D (1999). Epithelial sodium channel regulated by aldosterone-induced protein sgk. Proc Natl Acad Sci U S A.

[11] Maiyar AC, Huang AJ, Phu PT, Cha HH, Firestone GL (1996). p53 stimulates promoter activity of the sgk. Serum/glucocorticoid-inducible serine/threonine protein kinase gene in rodent mammary epithelial cells. J Biol Chem.

[12] Mizuno H, Nishida E (2001). The ERK MAP kinase pathway mediates induction of SGK (serum- and glucocorticoid-inducible kinase) by growth factors. Genes Cells.

[13] You H, Jang Y, You-Ten AI, Okada H, Liepa J, Wakeham A, Zaugg K, Mak TW (2004). p53-dependent inhibition of FKHRL1 in response to DNA damage through protein kinase SGK1. Proc Natl Acad Sci U S A.

[14] Brunet A, Park J, Tran H, Hu LS, Hemmings BA, Greenberg ME (2001). Protein kinase SGK mediates survival signals by phosphorylating the forkhead transcription factor FKHRL1 (FOXO3a). Mol Cell Biol.

[15] Lang F, Henke G, Embark HM, Waldegger S, Palmada M, Bohmer C, Vallon V (2003). Regulation of channels by the serum and glucocorticoid-inducible kinase - implications for transport, excitability and cell proliferation. Cell Physiol Biochem.

[16] Murray JT, Campbell DG, Morrice N, Auld GC, Shpiro N, Marquez R, Peggie M, Bain J, Bloomberg GB, Grahammer F (2004). Exploitation of KESTREL to identify NDRG family members as physiological substrates for SGK1 and GSK3. Biochem J.

[17] Melotte V, Qu X, Ongenaert M, van Criekinge W, de Bruine AP, Baldwin HS, van Engeland M (2010). The N-myc downstream regulated gene (NDRG) family: diverse functions, multiple applications. FASEB J.

[18] Chang X, Xu X, Ma J, Xue X, Li Z, Deng P, Zhang S, Zhi Y, Chen J, Dai D (2014). NDRG1 expression is related to the progression and prognosis of gastric cancer patients through modulating proliferation, invasion and cell cycle of gastric cancer cells. Mol Biol Rep.

[19] Mao Z, Sun J, Feng B, Ma J, Zang L, Dong F, Zhang D, Zheng M (2013). The metastasis suppressor, N-myc downregulated gene 1 (NDRG1), is a prognostic biomarker for human colorectal cancer. PLoS One.

[20] Dos Santos M, da Cunha Mercante AM, Nunes FD, Leopoldino AM, de Carvalho MB, Gazito D, Lopez RV, Chiappini PB, de Carvalho Neto PB, Fukuyama EE (2012). Prognostic significance of NDRG1 expression in oral and oropharyngeal squamous cell carcinoma. Mol Biol Rep.

[21] Nishio S, Ushijima K, Tsuda N, Takemoto S, Kawano K, Yamaguchi T, Nishida N, Kakuma T, Tsuda H, Kasamatsu T (2008). Cap43/NDRG1/Drg-1 is a molecular target for angiogenesis and a prognostic indicator in cervical adenocarcinoma. Cancer Lett.

[22] Sibold S, Roh V, Keogh A, Studer P, Tiffon C, Angst E, Vorburger SA, Weimann R, Candinas D, Stroka D (2007). Hypoxia increases cytoplasmic expression of NDRG1, but is insufficient for its membrane localization in human hepatocellular carcinoma. FEBS Lett.

[23] Fan C, Yu J, Liu Y, Xu H, Wang E (2012). Increased NDRG1 expression is associated with advanced T stages and poor vascularization in non-small cell lung cancer. Pathol Oncol Res.

[24] Ando T, Ishiguro H, Kimura M, Mitsui A, Kurehara H, Sugito N, Tomoda K, Mori R, Takashima N, Ogawa R (2006). Decreased expression of NDRG1 is correlated with tumor progression and poor prognosis in patients with esophageal squamous cell carcinoma. Dis Esophagus.

[25] Ai R, Sun Y, Guo Z, Wei W, Zhou L, Liu F, Hendricks DT, Xu Y, Zhao X (2016). NDRG1 overexpression promotes the progression of esophageal squamous cell carcinoma through modulating Wnt signaling pathway. Cancer Biol Ther..

[26] Brierley JD, Gospodarowicz MK, Wittekind C. TNM classification of malignant tumours. Hoboken: Wiley-Blackwell; 2016.

[27] Eisenhauer EA, Therasse P, Bogaerts J, Schwartz LH, Sargent D, Ford R, Dancey J, Arbuck S, Gwyther S, Mooney M (2009). New response evaluation criteria in solid tumours: revised RECIST guideline (version 1.1). Eur J Cancer.

[28] Japanese Classification of Esophageal Cancer, 11th Edition: part II and III. Esophagus. 2017;14(1):37–65. 10.1007/s10388-016-0556-2 PMC5222925 28111536

[29] Ozawa Y, Nakamura Y, Fujishima F, Felizola SJ, Takeda K, Okamoto H, Ito K, Ishida H, Konno T, Kamei T (2015). c-met in esophageal squamous cell carcinoma: an independent prognostic factor and potential therapeutic target. BMC Cancer.

[30] Sato N, Fujishima F, Nakamura Y, Aoyama Y, Onodera Y, Ozawa Y, Ito K, Ishida H, Kamei T, Watanabe M (2018). Myosin 5a regulates tumor migration and epithelial-mesenchymal transition in esophageal squamous cell carcinoma: utility as a prognostic factor. Hum Pathol.

[31] Xiaobo Y, Qiang L, Xiong Q, Zheng R, Jianhua Z, Zhifeng L, Yijiang S, Zheng J (2016). Serum and glucocorticoid kinase 1 promoted the growth and migration of non-small cell lung cancer cells. Gene..

[32] Gao S, Wang D, Kong G, Li S, Wang W, Wang H, Zhou F (2017). Expression of serum- and glucocorticoid-regulated kinase 1 and its association with clinicopathological factors and the survival of patients with adenocarcinoma of the esophagogastric junction. Oncol Lett.

[33] Liang X, Lan C, Jiao G, Fu W, Long X, An Y, Wang K, Zhou J, Chen T, Li Y (2017). Therapeutic inhibition of SGK1 suppresses colorectal cancer. Exp Mol Med.

[34] Rossi Luigi, Verrico Monica, Tomao Silverio, Ricci Fabio, Fontana Antonella, Spinelli Gian Paolo, Colonna Maria, Vici Patrizia, Tomao Federica (2019). Expression of ER, PgR, HER-2, and Ki-67 in core biopsies and in definitive histological specimens in patients with locally advanced breast cancer treated with neoadjuvant chemotherapy. Cancer Chemotherapy and Pharmacology.

[35] Kokame K, Kato H, Miyata T (1996). Homocysteine-respondent genes in vascular endothelial cells identified by differential display analysis. GRP78/BiP and novel genes. J Biol Chem.

[36] Kurdistani SK, Arizti P, Reimer CL, Sugrue MM, Aaronson SA, Lee SW (1998). Inhibition of tumor cell growth by RTP/rit42 and its responsiveness to p53 and DNA damage. Cancer Res.

[37] Mamada N, Tanokashira D, Ishii K, Tamaoka A, Araki W (2017). Mitochondria are devoid of amyloid beta-protein (Abeta)-producing secretases: evidence for unlikely occurrence within mitochondria of Abeta generation from amyloid precursor protein. Biochem Biophys Res Commun.

[38] Mattern J, Buchler MW, Herr I (2007). Cell cycle arrest by glucocorticoids may protect normal tissue and solid tumors from cancer therapy. Cancer Biol Ther..

[39] Zhang C, Kolb A, Mattern J, Gassler N, Wenger T, Herzer K, Debatin KM, Buchler M, Friess H, Rittgen W (2006). Dexamethasone desensitizes hepatocellular and colorectal tumours toward cytotoxic therapy. Cancer Lett.

[40] Mikosz CA, Brickley DR, Sharkey MS, Moran TW, Conzen SD (2001). Glucocorticoid receptor-mediated protection from apoptosis is associated with induction of the serine/threonine survival kinase gene, sgk-1. J Biol Chem.

[41] Wei W, Bracher-Manecke JC, Zhao X, Davies NH, Zhou L, Ai R, Oliver L, Vallette F, Hendricks DT (2013). Oncogenic but non-essential role of N-myc downstream regulated gene 1 in the progression of esophageal squamous cell carcinoma. Cancer Biol Ther.

[42] Zhang C, Mattern J, Haferkamp A, Pfitzenmaier J, Hohenfellner M, Rittgen W, Edler L, Debatin KM, Groene E, Herr I (2006). Corticosteroid-induced chemotherapy resistance in urological cancers. Cancer Biol Ther..

[43] Sommer EM, Dry H, Cross D, Guichard S, Davies BR, Alessi DR (2013). Elevated SGK1 predicts resistance of breast cancer cells to Akt inhibitors. Biochem J.

